# Experimental observation of asymmetrical microwave jets and far-field distribution generated by a dual-material system

**DOI:** 10.1038/s41598-021-91400-2

**Published:** 2021-06-04

**Authors:** B. Varghese, O. Shramkova, P. Minard, L. Blondé, V. Drazic, V. Allié

**Affiliations:** 1 InterDigital R&D France, 975 Avenue des Champs Blancs, 35576 Cesson Sévigné, France; 2 Technicolor Connected Home, 975 Avenue des Champs Blancs, 35576 Cesson Sévigné, France

**Keywords:** Optical physics, Optical physics

## Abstract

In this paper, we report the experimental and numerical investigation of plane wave diffraction by an all-dielectric dual-material cuboid. Edge diffraction by a cuboid leads to the generation of a narrow, high intensity beam in the near-field region called a photonic jet. We examine the dependence of the jet behavior and orientation on the materials and dimensions of constitutive parts in the microwave frequency domain. The possibility to shift and deviate the resultant microwave jet in the near-field region of such a structure depending on the size of constitutive parts is demonstrated numerically. Experimentally, we observe a shift in the spatial position of the jet. The experimental asymmetric electric field profile observed in the far-field region is attributed to the input of multiple edge waves generated by the dual-material cuboid. The presented results may be scaled at different frequency bands such as optical frequencies for designing nanostructures enabling the focusing and deviation functionality and creation of new optical devices which would satisfy the needs of emerging nanophotonic applications.

## Introduction

A photonic jet is a narrow, high-intensity electromagnetic beam formed on the shadow side surface of an element illuminated by a plane wave. The concept was first reported by modelling the response of micron-scale dielectric cylinders illuminated by a plane wave^1^. Due to small scale of the structures involved, they were termed as nanojets. The formalism of photonic nanojets for dielectric microspheres^2^ and microcylinders^3^ can be understood using Mie’s theory. A detailed literature review on photonic nanojets has been reported by Heifetz et al.^4^. Jets of the same nature have also been reported with mesoscale objects irradiated by THz frequencies in which case the jets are termed as terajets^5–7^. Heifetz et al.^8^ report an enhanced backscattering by a jet created by a dielectric sphere of diameter 7.62 cm at 30 GHz. Kong et al.^9^ created a microwave jet by a 5.08 cm acrylic sphere illuminated by plane polarized EM waves of 30 GHz frequency. Minin et al.^7^ experimentally observe a photonic hook phenomenon (a type of curved light beam created by the focusing of a plane wave through an asymmetric dielectric particle^10^) at 0.25 THz frequency using a mesoscale asymmetric dielectric cuboid. Pacheco-Pena et al.^5^ experimentally studied terajets created from dielectric cuboids at sub-THz frequencies. Creation of photonic jets by dielectric particles of different shapes have also been reported in the literature^11,12^.

Microwave jets^9^, generated in the microwave frequency domain are analogues of photonic nanojets. Recently, studies have shown how edge diffraction phenomenon can explain the focusing properties of arbitrary shaped microstructures^13,14^. Diffraction of an incident plane wave from the edge of a dielectric microstructure forms a tilted focused beam (a.k.a. photonic jet) whose deviation angle depends on the refractive index ratio between the structure medium and the surrounding material. Design of metagrating diffractive elements for waveguide in-couplers^15^ and color splitting elements for image sensors^16^ using photonic jet combination by dual-material elements have previously been demonstrated.

**Figure 1:**
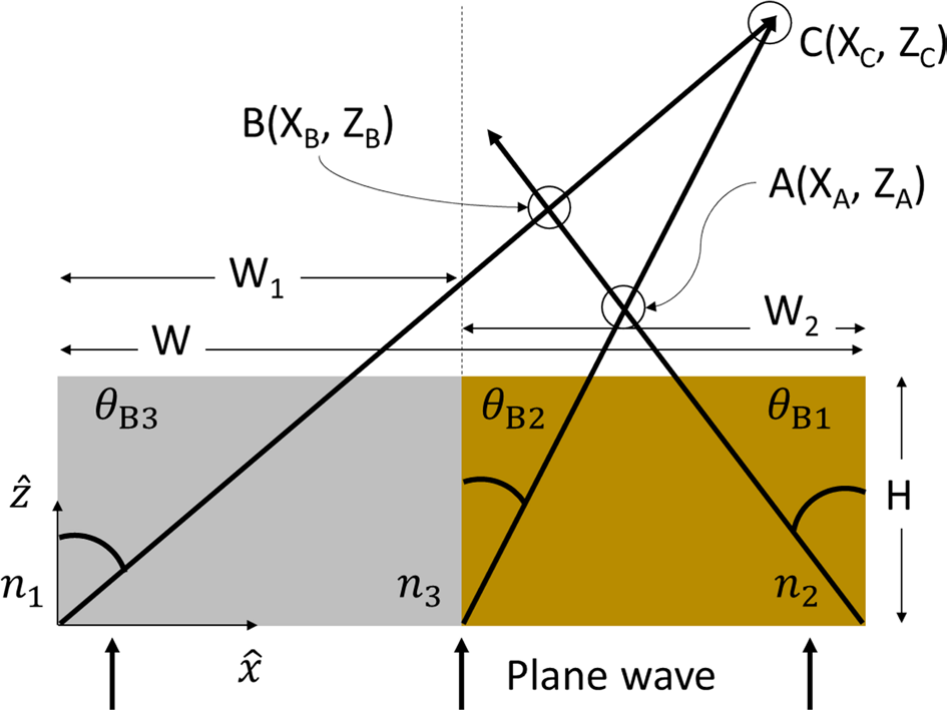
Schematic representation of the cross-sectional geometry of a dual-material cuboid illuminated by a plane wave. The arrows indicate the direction of the photonic jets created and their position of intersection.

The photonic jet beam angle for edges with a base angle of 90° (refer Fig. 1) can be approximated by the following relation^13^:

1 $$\theta_{{{\text{B}}j}} \cong \frac{{90^\circ - {\uptheta }_{{{\text{TIR}}j}} }}{2}$$

where *j* = 1, 2, 3 and ${\uptheta }_{{{\text{TIR}}1}} = \sin^{ - 1} \left( {\frac{{{\text{n}}_{1} }}{{{\text{n}}_{2} }}} \right),{\uptheta }_{{{\text{TIR}}2}} = \sin^{ - 1} \left( {\frac{{{\text{n}}_{3} }}{{{\text{n}}_{2} }}} \right),{\uptheta }_{{{\text{TIR}}3}} = \sin^{ - 1} \left( {\frac{{{\text{n}}_{1} }}{{{\text{n}}_{3} }}} \right)$ are the critical angles of total internal reflection. The angles of each photonic jet deviation depend on the ratio of indexes of the two media in contact. The maximal intensity and minimal length correspond to the beam with the highest ratio between the refractive indexes in the expression for ${\uptheta }_{{{\text{TIR}}j}}$. Each photonic jet is a result of constructive interference between the transmitted plane wave and an edge wave generated at the edge of an interface separating two mediums^14^. It makes approximately twice the angle of the photonic jet i.e. $\theta_{{\text{Edge Wave}}} = 2\theta_{Bj}$ w.r.t. the vertical. Influence of edge waves on the far-field (FF) response of a system is observed in the following sections.

For the case of a single-material system with vertical edges (*n*_3_ = *n*_2_ in Fig. 1), photonic jets radiate from opposite edges with $\theta_{B3} = \theta_{B1}$, intersect and combine on the vertical axis of symmetry. To understand the behavior of the combined photonic jet radiated by a dual-material structure with constitutive material of widths W_1_ and W_2_ as shown in Fig. 1 (W = W_1_ + W_2_), we must analyze the individual photonic jet orientation and determine the condition for their intersections and their positions. In Fig. 1, point A denotes the point of intersection of jet 1 (with an angle of *θ*_*B*1_) and jet 2 (with an angle of *θ*_*B*2_) having the coordinates (X_A_, Z_A_), where

2 $$\begin{aligned} X_{A} & = {\text{W}}_{1} + \tan \left( {\theta_{B2} } \right)*Z_{A} , \\ Z_{A} & = \frac{{W_{2} }}{{{\text{tan}}\left( {\theta_{B1} } \right) + {\text{tan}}\left( {\theta_{B2} } \right)}}. \\ \end{aligned}$$

The jets generated by the external vertical edges of the dual-material system (jet1 with angle *θ*_*B*1_ and jet 3 with an angle *θ*_*B*3_) will intersect at point B with the coordinates (X_B_, Z_B_), where

3 $$\begin{aligned} X_{B} & = \tan \theta_{B3} *Z_{B} , \\ Z_{B} & = \frac{W}{{{\text{tan}}\left( {\theta_{B1} } \right) + {\text{tan}}\left( {\theta_{B3} } \right)}}. \\ \end{aligned}$$

Using Eq. (1), we note that jet 2 and jet 3 would intersect only when $n_{3} \ge \sqrt {n_{1} n_{2} }$ in which case the coordinates of point C (X_C_, Z_C_) would be

4 $$\begin{aligned} X_{C} & = \tan \theta_{B3} *Z_{C} , \\ Z_{C} & = \frac{{W_{1} }}{{{\text{tan}}\left( {\theta_{B3} } \right) - {\text{tan}}\left( {\theta_{B2} } \right)}}. \\ \end{aligned}$$

Numerical analysis of the power density distribution inside and outside the element demonstrates that the position of photonic jet hotspot and shape of generated beam are determined by the sizes of the blocks. For systems with equal sizes of constitutive parts (i.e. *W*_1_ = *W*_2_), for W/2 > *λ* and $n_{3} < \sqrt {n_{1} n_{2} }$.

I. If *H* < *Z*_*A*_, the photonic jet shifts from the axis of symmetry toward the higher refractive index medium^17^,
II. If *H* > *Z*_*A*_, the photonic jet deviates towards the medium with refractive index n_2_ (n_2_ > n_3_).

For *W*/2 < *λ* we observe a shift of the photonic jet towards the part with higher refractive index. For our simulations we chose *W*_1_ = *W*_2_ = 6 cm for incident frequencies of 4, 5 and 6 GHz (or *λ* = 7.5, 6 and 5 cm, respectively). For 4 GHz we have *W*/2 < *λ*, so we should observe a shift of the combined microwave jet, whereas for 6 GHz, *W*/2 > *λ* and so we should see a deviation of the jet orientation (for H > Z_A_). The parameters used were taken with the understanding that the photonic jet phenomenon is scalable with wavelength and keeping in mind the experimental aspect of the study. The materials we used were low-loss foam dielectric cuboids with *ε*_*r*_ = 2.6 and 3.2 (*n* = 1.61 and 1.79 respectively). *Z*_*A*_ for these materials was calculated to be 7.9 cm. To observe the deviation, we first simulated a system with the height *H* = 8.5 cm, total width *W* = 12 cm and depth *D* = 12 cm. The jet created by the dual-material cuboid was compared to that by a single-material cuboid with an isotropic *ε*_*r*_ = 2.6 having the same physical dimensions. Table 1 summarizes the dielectric cuboid parameters used in this study.

**Table 1:** Summary of details of the dielectric cuboids used in this study.

Cuboid type	Relative permittivity, *ε*_*r*_	Loss tangent	Total width, W (cm)	Depth, D (cm)	Height, H (cm)
Single material	2.6	0.0009	12	12	8.5
Dual-material	2.6 and 3.2	0.0009 and 0.0010	12 (6 + 6)	12 (each)	8.5 (each)

## Results and discussion

This section consists of numerical and experimental results in the NF and FF domain. The methodology used is detailed in the Methods section later in the manuscript.

### Numerical results

**Figure 2:**
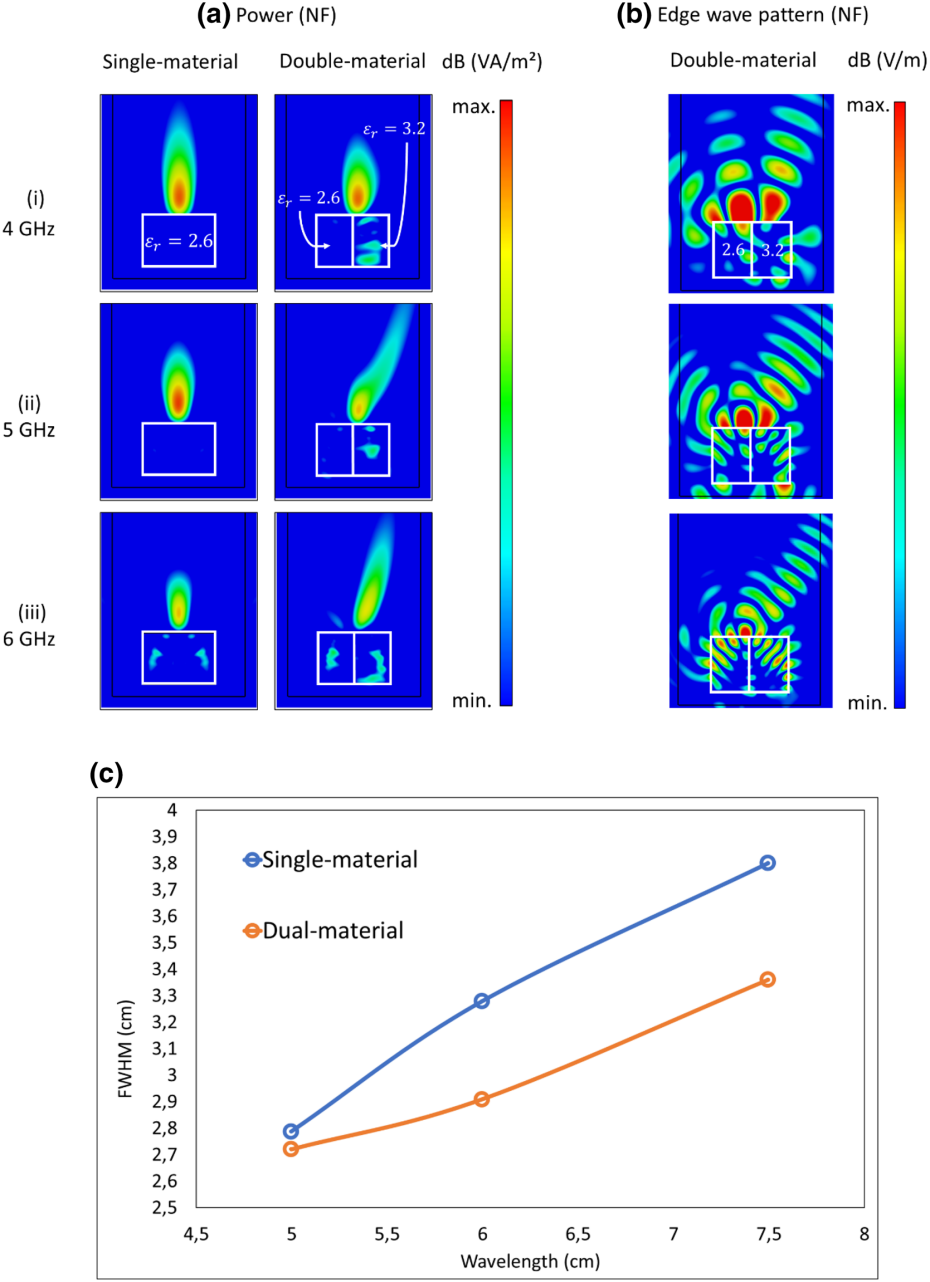
Numerical simulations of power (**a**) for single- and dual-material systems, and edge wave pattern (**b**) for dual-material systems at three different incident frequencies of 4 (i), 5 (ii) and 6 (iii) GHz. The incident wave illuminates the systems from the bottom. (c) FWHM of the microwave jets at different incident wavelengths.

Figure 2a shows the spatial distribution of power in the near-field (NF) for the three frequencies considered. As mentioned previously, constructive interference between the transmitted waves and the edge waves (refer Fig. 2b) results in a single combined microwave jet generated by the dielectric cuboids that appear as EM field hotspots as shown. Due to proper choice of the material refractive index ratios and structural dimensions, the microwave jets are created outside the dielectric cuboids in air where they could be experimentally detected. The intensity of generated beam varies as the incident frequency changes showing a dependence on the structure height relative to wavelength. Due to symmetry of the system, for the case of a single-material cuboid, the microwave jets produced by opposite edges of the cuboid combine to form one single jet along the axis of symmetry of the system for all frequencies. For the case of dual-material cuboid, owing to relative permittivity difference of constituent materials of the system, the generated microwave jet slightly shifts toward the higher permittivity medium for 4 GHz incidence, while it tilts away from the vertical axis towards the higher permittivity material for 5 and 6 GHz incident waves. Figure 2c shows the numerically determined Full-Width-Half-Maximum (FWHM) values at the microwave jet maximum. It is seen that the microwave jet for the dual-material system is thinner than its single-material counterpart for all considered frequencies (or corresponding wavelengths) with a tendency to widen as the incidence wavelength increases.

### Experimental results

**Figure 3:**
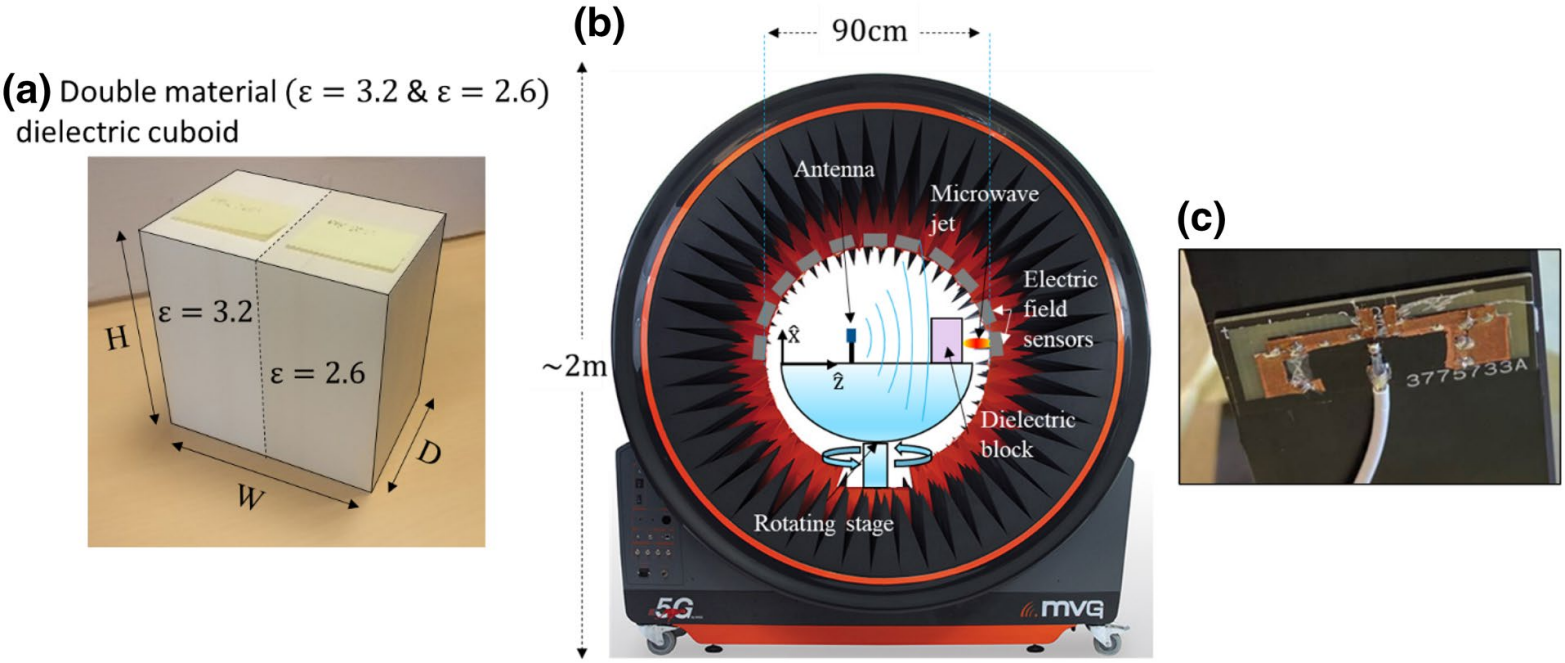
(**a**) Dual-material dielectric cuboid with relative permittivities of *ε*_*r*_ = 3.2 and *ε*_*r*_ = 2.6 The physical dimensions are H = 8.5 cm, W = 12 cm, D = 8.5 cm. (**b**) Snapshot of the *STARlab* apparatus with a schematic of the set-up. (c) Snapshot of the electric dipole source antenna used in this study.

Prototypes with the parameters mentioned in Table 1 were fabricated. The single-material dielectric cuboid prototype is labelled P1 whereas the dual-material one is labelled P2 henceforth. Figure 3a–c show snapshots of the dual-material dielectric cuboid, the experimental apparatus and the electric dipole source antenna used to characterize the response of the system in this study, respectively.

**Figure 4:**
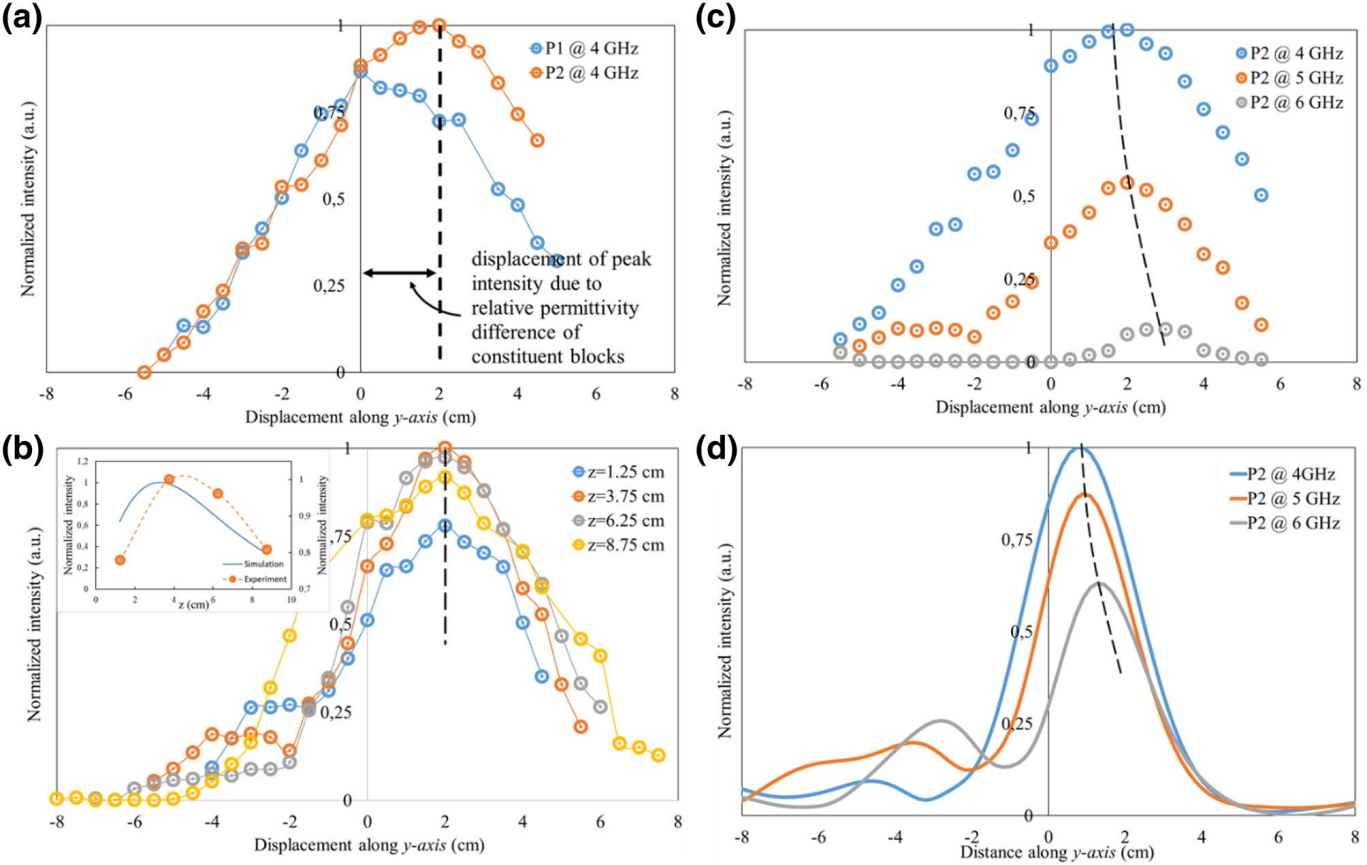
Experimental NF electric field intensity measurements (**a**) along y-axis for prototypes P1 and P2 at a fixed separation distance of *z* = 3.75 cm from the sensor for illumination frequencies of 4 GHz, (**b**) along y-axis for just prototype P2 at 5 GHz for different separation distances, *z* (inset: EM field intensity variation along z-axis measured experimentally (orange curve) and numerically (blue curve) at y = 2 cm. (c, d) Comparison of NF intensities for P2 at illumination frequencies of 4, 5 and 6 GHz for experiments (c) and simulations (d).

Figure 4a is an experimental measurement of electromagnetic field intensity (y) at the shadow side of prototypes P1 and P2 kept at a fixed distance of *z* = 3.75 cm from the sensor and illuminated by plane waves of 4 GHz. The intensity values are normalized according to the following relation:

5 $${\text{y}}_{{{\text{normalized}}}} = \frac{{{\text{y}} - {\text{y}}_{{{\text{min}}}} }}{{{\text{y}}_{{{\text{max}}}} - {\text{y}}_{{{\text{min}}}} }}$$

where y_min_ and y_max_ are the minimum and maximum values of the electromagnetic field intensity, respectively. We observe that for both prototypes the intensity profile is a bell-curve confirming the generation of a microwave jet with the maximum for P1 along the vertical axis of symmetry of the system while that of P2 shifted by a certain distance. This shift of the peak position is attributed to relative permittivity difference of the constituents of P2 and is coherent with Fig. 2a(i). Figure 4b demonstrates the electromagnetic field intensity profile for P2 at 5 GHz incidence for different separation distances from the sensor. The inset of Fig. 4b shows the EM field intensity variation along $\hat{z}$ of the microwave jet hotspot peak at y = 2 cm measured experimentally (orange curve) and numerically (blue curve) as a function of the separation distance. We experimentally observe the hotspot maximum to be at a distance of z = 3.75 cm from the system which is close to that expected from simulations (z = 3.3 cm). Moreover, the trend of width of the intensity curves at different positions of z is also coherent with the simulations. Figure 4c, d compare the experimental (top) measurement of microwave jet intensity for P2 for three different frequencies at a separation distance of z = 3.75 cm with corresponding simulations (bottom) at a separation distance of 2 cm. The reason for this discrepancy in the separation distance is attributed to experiment imperfections. Nonetheless, we are still in the microwave jet region to say the behavior at each frequency is coherent with what is expected from simulations. We observe the microwave jet for 4 GHz frequency is the strongest which then drops monotonically with the incident frequency.

**Figure 5:**
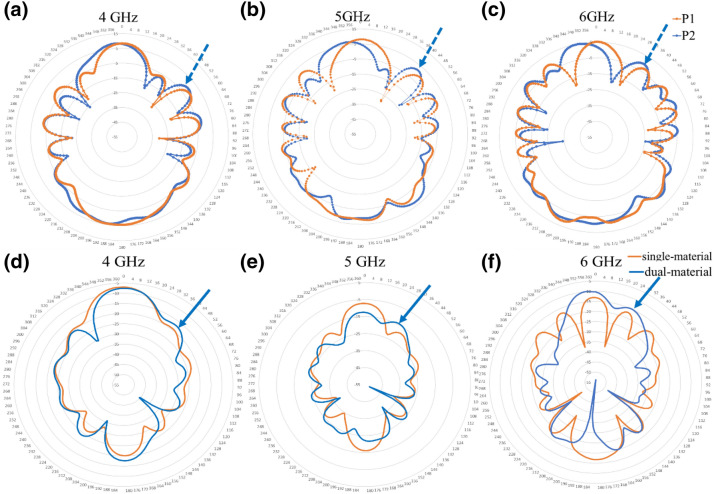
Experimental (**a**–**c**) FF electric field radiation pattern for prototypes P1 (orange) and P2 (blue) with numerically simulated FF radiation pattern (**d**–**f**) for single-material (orange) and dual-material (blue) systems at illumination frequencies of 4, 5 and 6 GHz.

Figure 5 demonstrates the experimentally measured electric field response in the FF domain for prototypes P1 (orange curves) and P2 (blue curves) at illuminating frequencies of 4 GHz (Fig. 5a), 5 GHz (Fig. 5b) and 6 GHz (Fig. 5c), respectively. According to antenna theory, the criterion of FF region starting from $\frac{2D}{\lambda }$ where, D is the maximum linear dimension of the antenna (= 3 cm), leads to a value of 2.4 cm for 4 GHz, 3 cm for 5 GHz and 3.6 cm for 6 GHz. For prototype P1 (single-material cuboid), due to the symmetry of the physical system, we detect a symmetric FF profile of the electric field radiation with a strong principal lobe oriented along the axis of symmetry, irrespective of the structure size to wavelength ratio (W/(2λ)). However, for the case of prototype P2 (dual-material cuboid), due to relative permittivity difference of the constituent materials, we observe an asymmetry in the response of the electric field radiation pattern in the FF primarily manifested as a deviated principal lobe and a stronger secondary lobe on the higher permittivity material side. The degree of deviation of the principal lobe increases with W/(2λ). This asymmetric behavior seen for the dual-material system is coherent with the numerically simulated response shown in Fig. 5d–f. We observe a symmetric E-pattern for the single-material cuboid (orange curves) having its principal lobe pointing in the direction of propagation for the considered incident frequencies. This is an expected behavior considering the symmetrical nature of the system. However, for the dual-material cuboid (blue curves) the pattern gets asymmetric:

- For 4 GHz: simulations (Fig. 5d) predict a principal lobe pointing towards the vertical direction and a secondary lobe at ~45° (on the higher permittivity material side), marked with a solid blue arrow, stronger in amplitude than the corresponding secondary lobe for single material system. In the experimental measurements (Fig. 5a) we see the principal lobe pointing along the vertical with a secondary lobe at ~50° (on the higher permittivity material side), marked with a dashed blue arrow, stronger than the corresponding lobe for the single material system.
- For 5 and 6 GHz: simulations (Fig. 5e, f) predict an asymmetric FF profile with the principal lobes being tilted slightly towards the lower permittivity material side (left) and a secondary lobe on the higher permittivity material side at ~28°, marked by blue solid arrows, stronger than that of the single material system. In the experimental measurements (Fig. 5b, c), we observe an asymmetric FF profile with their principal lobes tilted slightly towards the lower permittivity material side (left) and a secondary lobe at ~40° for the 5 GHz and ~35° for 6 GHz, marked by blue dashed arrows.

We attribute this asymmetric FF behavior of the lobes to the input of three edge waves generated by the vertical edges of the system as can be seen in Fig. 2b for the edge wave pattern for different incident frequencies.

## Conclusion

In conclusion, an experimental generation of a microwave photonic jet and the possibility to spatially shift its position in the NF zone was confirmed with a dual-material structure illuminated by a plane wave in the microwave domain. A microwave jet is the result of constructive interference between the transmitted plane wave and an edge wave generated by the edges of two separate media. By varying the relative structure size and relative permittivity, we can change the behavior of the combined edge wave and the resultant microwave jet. We have examined the dependence of microwave jet behavior and orientation on the materials, frequency, and dimensions of the constitutive parts in the NF. The asymmetric FF electric field pattern observed experimentally for a dual-material system is linked with an asymmetric orientation of the combined edge wave generated by the element. The system proposed here could be scaled down to be used at optical frequencies with the results potentially contributing to the development of a new type of optical manipulation device for trapping or moving micro/nanoparticles or help in designing diffraction grating elements.

## Methods

The electromagnetic responses of the single-material and dual-material systems were numerically simulated using the time-domain solver of commercially available software CST Microwave Studio. Transverse Magnetic (TM) polarized plane wave propagating along $\hat{z}$(refer Fig. 1) illuminated the system immersed in air medium bounded by perfectly matched layers to absorb undesired back reflections by the boundaries. At the cuboid’s shadow side, an additional space of 6*λ* was added to properly visualize the resulting photonic jet characteristics.

To experimentally observe the microwave jets for the simulated cases, we fabricated the dielectric cuboids with the parameters mentioned in Table 1. The prototypes were made from solid *PREPERM* foams with the respective permittivities at the required frequencies. The roughness of the cuboids used in this study were very low compared to the incident wavelengths to have negligible impact on EM wave scattering/loss. Microwave jet characteristics of prototypes P1 and P2 were experimentally measured using MVG group *STARLab* apparatus (refer Fig. 3b) typically used for antenna pattern and NF electric field measurements of devices. The far-field measurements were done by placing the system and source in the center of *STARLab* apparatus on the rotating stage which then rotated in the y–z plane measuring the FF electric field intensity. The apparatus is a small anechoic chamber 90 cm in diameter consisting of a graduated rotating stage on a rigid mast. The arching walls of the apparatus consist of bipolarized Vivaldi antennas as probes with an angular spacing of 22.5° between two probes, each capable of measuring the vertical and horizontal components of electric field. For our NF study, only the sensor straight in front of the system was utilized. The source of incident EM waves used in the experiment to illuminate the single- and dual-material system was a Wi-Fi dual-band electric dipole antenna, 3 cm × 1.2 cm in size capable of emitting radiations of 4 GHz to 6 GHz. The theory of microwave jet formation requires a plane wave illuminating an interface. Since the source antenna emits spherical waves, it was necessary to place it at a sufficiently far away distance from the dielectric blocks to make sure that the waves that hit it are quasi-planar, not spherical. Graduations on the stage permit displacement of the cuboids and the measurement of its influence on the generated microwave jet characteristics. The prototypes were studied one at a time for each frequency. Using this set-up, we were able to study/measure the NF and 3D FF radiation pattern of the two dielectric cuboids successfully.

## Data Availability

The datasets generated during and/or analysed during the current study are available from the corresponding author on reasonable request.

## References

[1] Chen Z, Taflove A, Backman V (2004). Photonic nanojet enhancement of backscattering of light by nanoparticles: A potential novel visible-light ultramicroscopy technique. Opt. Express.

[2] Li X, Chen Z, Taflove A, Backman V (2005). Optical analysis of nanoparticles via enhanced backscattering facilitated by 3-D photonic nanojets. Opt. Express.

[3] Luk’yanchuk BS, Paniagua-Domínguez R, Minin I, Minin O, Wang Z (2017). Refractive index less than two: photonic nanojets yesterday, today and tomorrow [Invited]. Optical Materials Express.

[4] Heifetz A, Kong SC, Sahakian AV, Taflove A, Backman V (2009). Photonic nanojets. J. Comput. Theor. Nanosci..

[5] Pacheco-Peña V, Beruete M, Minin IV, Minin OV (2014). Terajets produced by dielectric cuboids. Appl. Phys. Lett..

[6] Pacheco-Peña V, Beruete M, Minin IV, Minin OV (2015). Multifrequency focusing and wide angular scanning of terajets. Opt. Lett..

[7] Minin IV (2019). Experimental observation of a photonic hook. Appl. Phys. Lett..

[8] Heifetz A (2006). Experimental confirmation of backscattering enhancement induced by a photonic jet. Appl. Phys. Lett..

[9] Kong SC, Sahakian AV, Heifetz A, Taflove A, Backman V (2008). Robust detection of deeply subwavelength pits in simulated optical data-storage disks using photonic jets. Appl. Phys. Lett..

[10] Yue L (2018). Photonic hook: a new curved light beam. Opt. Lett..

[11] Geints YE, Zemlyanov AA, Panina EK (2016). Photonic nanojets as a versatile optical tool for wave super-localization. AIP Conf. Proc..

[12] Minin IV, Minin OV, Geints YE (2015). Localized em and photonic jets from non-spherical and non-symmetrical dielectric mesoscale objects: Brief review. Ann. Phys..

[13] Boriskin A (2018). Near field focusing by edge diffraction. Opt. Lett..

[14] Varghese, B., Shramkova, O., Drazic, V., Allie, V. & Blonde, L. Influence of an edge height on the diffracted em field distribution. in *21st International Conference on Transparent Optical Networks (ICTON 2019)* (eds. Jaworski, M. & Marciniak, M.) 1135–1138 (IEEE, 2019). 10.1109/ICTON.2019.8840487.

[15] Shramkova, O., Drazic, V., Blondé, L., Varghese, B. & Allié, V. Nanojet-based dielectric multimaterial color splitters for image sensor applications. in *Proceedings of the SPIE 11368, Photonics and Plasmonics at the Mesoscale*, Vol. 1136809 (2020). 10.1117/12.2555400.

[16] Shramkova, O., Drazic, V., Blondé, L., Varghese, B. & Allié, V. High-Uniformity , High-Performance Double Material Dielectric Diffractive Metagratings. in *14th International Congress on Artificial Materials for Novel Wave Phenomena—Metamaterials 2020* vol. 3 3–5 (2020).

[17] Shramkova, O., Blondé, L., Drazic, V., Varghese, B. & Allié, V. Photonic nanojet generated by dielectric multi-material. in *Proceedings of the10th International Conference on Metamaterials, Photonic Crystals, Plasmonics, Meta 2019, Lisbon-Portugal* 899 (2019).

